# Prolonged venous transit is associated with worse neurological recovery in successfully reperfused large vessel strokes

**DOI:** 10.1002/acn3.52243

**Published:** 2024-11-11

**Authors:** Janet Mei, Hamza Adel Salim, Dhairya A. Lakhani, Licia Luna, Aneri Balar, Mona Shahriari, Nathan Z. Hyson, Francis Deng, Adam A. Dmytriw, Adrien Guenego, Vaibhav Vagal, Victor C. Urrutia, Elisabeth B. Marsh, Hanzhang Lu, Risheng Xu, Rich Leigh, Dylan Wolman, Gaurang Shah, Benjamin Pulli, Kambiz Nael, Gregory W. Albers, Max Wintermark, Jeremy J. Heit, Tobias D. Faizy, Argye E. Hillis, Raf Llinas, Vivek Yedavalli

**Affiliations:** ^1^ Division of Neuroradiology, Department of Radiology Johns Hopkins Medical Center Baltimore Maryland USA; ^2^ Department of Neuroradiology MD Anderson Medical Center Houston Texas 77030 USA; ^3^ Neuroendovascular Program, Massachusetts General Hospital Harvard University Boston Massachusetts USA; ^4^ Department of Medical Imaging, Neurovascular Centre St. Michael's Hospital Toronto Ontario Canada; ^5^ Department of Neurosurgery, Neurovascular Centre St. Michael's Hospital Toronto Ontario Canada; ^6^ Department of Diagnostic and Interventional Neuroradiology Erasme University Hospital Brussels Belgium; ^7^ Renaissance School of Medicine at Stony Brook University Stony Brook New York USA; ^8^ Department of Radiology Brown University Providence Rhode Island USA; ^9^ Department of Radiology, Division of Neuroradiology University of Michigan Ann Arbor Michigan USA; ^10^ Department of Interventional Neuroradiology Stanford Medical Center Palo Alto California USA; ^11^ Department of Radiology & Biomedical Imaging University of California San Francisco California USA; ^12^ Department of Radiology, Neuroendovascular Program University Medical Center Münster Germany

## Abstract

**Objective:**

Venous outflow (VO) impairment predicts unfavorable outcomes in patients with acute ischemic stroke caused by large vessel occlusion (AIS‐LVO). Prolonged venous transit (PVT), a visual qualitative VO marker on CT perfusion (CTP) time to maximum (Tmax) maps, has been associated with unfavorable 90‐day functional outcomes despite successful reperfusion. This study investigates the association between PVT and percent change on the National Institutes of Health Stroke Scale (NIHSS) among AIS‐LVO patients who have undergone successful reperfusion.

**Methods:**

We performed a retrospective analysis of prospectively collected data from consecutive adult AIS‐LVO patients with successful reperfusion (modified Thrombolysis in Cerebral Infarction 2b/2c/3). PVT+ was defined as Tmax ≥10 s in the superior sagittal sinus, torcula, or both. The primary outcome was continuous NIHSS percent change and dichotomous NIHSS percent change ≥70%. Regression analyses were performed to assess the effect of PVT on NIHSS percent change.

**Results:**

In 119 patients of median (IQR) age 71 (63–81) years, the admission and discharge NIHSS scores were significantly higher in PVT+ patients compared to PVT− patients (17 [14–23.5] vs. 13 [9.5–19], *p* = 0.011, and 7.5 [4–12] vs. 3 [1–7], *p* < 0.001, respectively). After adjusting for age, sex, hypertension, diabetes, atrial fibrillation, administration of intravenous thrombolysis (IVT), Alberta Stroke Program Early CT Scores (ASPECTS), mTICI 2c and/or 3, Tmax >6 s volume, and hemorrhagic transformation, PVT+ was significantly associated with lower NIHSS percent change (*B* = −0.163, 95%CI −0.326 to −0.001, *p* = 0.049) and was less likely to achieve higher than 70% NIHSS improvement (OR = 0.331, 95% CI 0.127–0.863, *p* = 0.024).

**Interpretation:**

PVT+ was significantly associated with reduced neurological improvement despite successful reperfusion in AIS‐LVO patients, highlighting the critical role of VO impairment in short‐term functional outcomes. These findings further validate PVT as a valuable adjunct imaging biomarker derived from CTP for assessing VO profiles in AIS‐LVO.

## Background

Venous outflow (VO) has emerged as an important imaging biomarker for prognosticating outcomes in patients with acute ischemic stroke due to large vessel occlusion (AIS‐LVO). Research has shown that unfavorable VO profiles are associated with a higher risk of cerebral edema,^1^, ^2^, ^3^ progression of edema,^4^, ^5^ hemorrhagic transformation,^6^ and poor clinical outcomes,^1^, ^7^, ^8^, ^9^, ^10^ even after successful reperfusion efforts. Conversely, favorable VO has been shown to predict better clinical outcomes, particularly in patients undergoing intravenous thrombolysis or mechanical thrombectomy.^11^, ^12^, ^13^

Prolonged venous transit (PVT) is defined as the presence of a time to maximum (Tmax) of ≥10 seconds in the superior sagittal sinus (SSS), the torcula, or both. PVT serves as a surrogate marker for VO status, integrating information from both superficial and deep cerebral venous drainage systems. The maker is readily interpretable on post‐processed Tmax maps from CT perfusion imaging. PVT has demonstrated utility by showing an association with unfavorable 90‐day mRS in successfully reperfused AIS‐LVO patients.^14^, ^15^ However, its prognostic significance concerning changes in neurological deficits over time from baseline has not been thoroughly investigated.

The National Institutes of Health Stroke Scale (NIHSS) is commonly used to quantify stroke deficits over time, starting from presentation. It offers insight beyond standard functional measures by tracking neurological improvement as an outcome. It is considered a reliable predictor of hospital length of stay, functional outcome at discharge, discharge disposition, and in‐hospital mortality.^16^, ^17^, ^18^, ^19^ Compared to absolute NIHSS score change, the NIHSS percent change offers a more accurate measurement by expressing change relative to the admission NIHSS score.^20^

This study aims to explore the relationship between PVT and NIHSS percent change in AIS‐LVO patients who have undergone successful reperfusion. We hypothesize that PVT+ is associated with a reduced likelihood of neurological improvement at discharge compared to PVT−.

## Methods

### Population

In this prospectively collected, retrospectively analyzed multicenter study, we identified patients with AIS with confirmed anterior circulation LVOs on CT angiography (CTA) using baseline comprehensive CT evaluation, which includes non‐contrast CT (NCCT), CTA, and CTP, treated at three centers between January 9, 2017 and September 22, 2022. Inclusion criteria were as follows: (1) AIS due to CTA‐confirmed LVO within 24 h of symptom onset; (2) available diagnostic CTP; (3) successful reperfusion after receiving MT with or without IVT, defined as modified Thrombolysis in Cerebral Infarction (mTICI) score of 2b/2c/3 on cerebral angiography; and (4) available NIHSS score at admission and discharge.

### Clinical data collection

Baseline clinical data collected for each patient included: demographics; risk factors for AIS (diabetes mellitus, hypertension, and atrial fibrillation); NIHSS score at admission and discharge; and length of hospital stay. NIHSS change (∆NIHSS) was defined as admission minus discharge NIHSS. NIHSS percent change was calculated using the formula: (∆NIHSS/admission NIHSS) × 100. A 70% cutoff was selected, as NIHSS percent change more than 70% has been demonstrated to predict favorable functional outcomes, defined as a modified Rankin Scale score of 0–2 at 90‐day follow‐up.^21^

In accordance with institutional protocols, the decision to treat with MT was made with the consensus of the multidisciplinary stroke team. If the patient was treated with MT, the thrombectomy technique (i.e., aspiration only, stent‐retriever only, or combination) and devices used were at the discretion of the neurointerventionalist. The final posttreatment mTICI score was determined by the treating neurointerventionalist and verified retrospectively by two expert consensus reviews. The discharge mRS scores were calculated by certified neurologists or nurse practitioners.

### Imaging data collection

Imaging data obtained from contemporaneous radiology reports were retrospectively validated by an experienced neuroradiologist. Post‐processed or otherwise retrospectively evaluated imaging data were performed by an experienced neuroradiologist. Alberta Stroke Program Early CT Scores (ASPECTS) were calculated from NCCTs. The presence of LVO, the segment of occlusion, and the laterality of the occlusion were identified based on baseline CTAs independently by experienced board‐certified neuroradiologists (VY, 10 years of working experience and DL, 4 years of working experience). Any discrepancies were resolved by consensus review.

### CT perfusion‐based assessment

Whole brain pretreatment CTP was performed on the Siemens Somatom Force (Erlangen, Germany) with the following parameters: 70 kVP, 200 effective mAs, rotation time 0.25 s, average acquisition time 60 s, collimation 48 × 1.2 mm, pitch value 0.7, 4D range 114 mm × 1.5 s. CTP images were then post‐processed using RAPID commercial software (IschemaView, Menlo Park, California, USA) for generating quantitative relative cerebral blood flow (rCBF) volumes, Tmax volumes, and qualitative Tmax maps.

**Figure 1:**
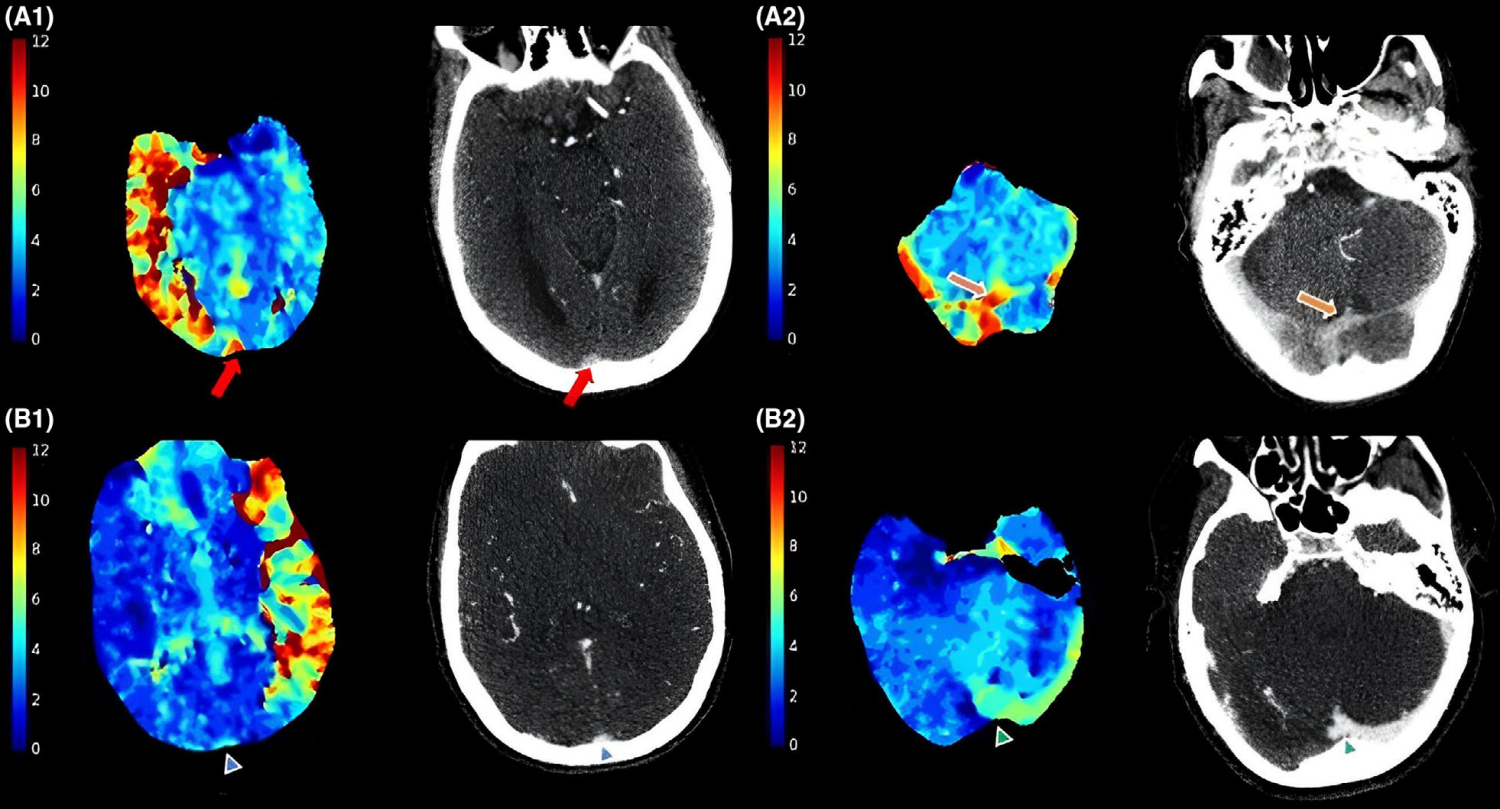
(A and B) PVT assessment in post‐processed Tmax maps (left) and corresponding axial CTA (right) views of two patients. (A1 and A2) A 64‐year‐old patient presented with a right internal carotid artery occlusion and admission NIHSS score of 18, and then discharged with NIHSS percent change of 61% at discharge. This patient was PVT+ as Tmax ≥10 s was observed in the posterior superior sagittal sinus at the level of the occipital horns (A1, red arrow) and at the torcula (A2, orange arrow). (B1 and B2) A 64‐year‐old patient presented with a left M1 middle cerebral artery occlusion and admission NIHSS score of 8, and then discharged with NIHSS percent change of 100%. This patient was PVT− as Tmax ≥10 s was not present either in the posterior superior sagittal sinus (B1, blue arrowhead) or at the torcula (B2, green arrowhead). CTA, CT angiography; NIHSS, National Institutes of Health Stroke Scale; PVT, prolonged venous transit; Tmax, time to maximum.

Based on qualitative Tmax maps, PVT was assessed within the posterior SSS at the level of the lateral ventricle occipital horns for more proximal venous drainage and at the torcula for deep venous drainage, by experienced board‐certified neuroradiologist (VY, 10 years of working experience and DL, 4 years of working experience). PVT+ was defined as Tmax ≥10 s timing within the posterior SSS, torcula, or both areas, and PVT− was defined as lacking Tmax ≥10 s timing within the SSS and the torcula (Fig. 1).

### Primary outcomes and statistical analysis

The primary outcome was the NIHSS percent change at discharge. Continuous data were reported as medians and interquartile ranges (IQR) and categorical variables were reported as frequencies. Data were compared using Wilcoxon rank sum tests or chi‐squared tests as applicable. Multiple linear regression analysis was used to assess the effect of PVT+ on NIHSS percent change. Multivariable logistic regression analysis was applied to dichotomous NIHSS percent change ≥70%. *p* values ≤0.05 were considered significant. All statistical analyses were conducted using IBM SPSS statistics (Version 22.0, Chicago, IL, USA).

## Results

### Baseline characteristics of the patient cohort

A total of 119 patients of median age 71 (IQR 63–81) years with 71 (59.7%) females were included in the study (Table 1). The median admission NIHSS score was 15 (IQR 11–20) and the median ASPECTS score was 9 (IQR 8–10). The median ischemic core volume (rCBF <30%) was 0 (IQR 0–25) ml and 100 (84.0%) patients had an ischemic core volume less than 50 ml. Thirty‐seven (31.1%) patients received intravenous thrombolysis and all patients went through mechanical thrombectomies. The median mRS score at discharge was 4 (IQR 2–5). The median length of hospital stay was 7 (IQR 4–14) days. The median 90‐day mRS score was 2 (IQR 1–4).

**Table 1:** Patient characteristics according to venous outflow dichotomized by prolonged venous transit.

	Total	Favorable venous outflow (PVT−)	Unfavorable venous outflow (PVT+)	*p* value
	*N* = 119	*N* = 83	*N* = 36	
Age, years	71 (63–81)	70 (61–77.5)	72 (62.57–81.75)	0.254
Gender, female, *n* (%)	71 (59.7)	52 (62.7)	19 (52.8)	0.313
Race, *n* (%)				0.123
African American	42 (35.3)	24 (28.9)	18 (50.0)	
Caucasian	69 (58.0)	54 (65.1)	15 (41.7)	
Asian	3 (2.5)	2 (2.4)	1 (2.8)	
Other	5 (4.2)	3 (3.6)	2 (5.6)	
Comorbidities, *n* (%)				
Hypertension	95 (79.8)	63 (75.9)	32 (88.9)	0.105
Hyperlipidemia	61 (51.3)	43 (51.8)	18 (50.0)	0.856
Diabetes mellitus	33 (27.7)	22 (26.5)	11 (30.6)	0.650
Atrial fibrillation	55 (46.2)	39 (47.0)	16 (44.4)	0.798
Prior stroke or TIA	23 (19.3)	16 (19.3)	7 (19.4)	0.983
Tobacco use, *n* (%)	58 (49.2)	36 (43.9)	22 (61.1)	0.085
ASPECTS	9 (8–10)	9 (8–10)	10 (8–10)	0.764
Occlusion location, *n* (%)				0.697
ICA	10 (8.4)	6 (7.2)	4 (11.1)	
M1	85 (71.4)	61 (73.5)	24 (66.7)	
M2	24 (20.2)	16 (19.3)	8 (22.2)	
rCBF<30% volume, mL	0 (0–25)	0 (0–23)	6 (0–33.75)	0.807
Tmax>6 s volume, mL	108 (66–156)	101 (57–138)	120 (87–195.5)	0.002
IVT, *n* (%)	37 (31.1)	27 (32.5)	10 (27.8)	0.607
mTICI				0.482
2b	33 (27.7)	24 (28.9)	9 (25.0)	
2c	22 (18.5)	13 (15.7)	9 (25.0)	
3	64 (53.8)	46 (55.4)	18 (53.8)	
Hemorrhagic transformation, *n* (%)	52 (43.7)	36 (43.4)	16 (44.4)	0.914
Parenchymal hematoma type 2, *n* (%)	9 (7.6)	4 (4.8)	5 (13.9)	0.086
Length of stay, days	7 (4–14)	6 (4–12)	9 (6–18.25)	0.031
90‐day mRS	2 (1–4)	2 (1–4)	4 (2–6)	<0.001

Values are represented as median (interquartile range) or number (percentage).

ASPECTS, Alberta Stroke Program Early CT Score; ICA, internal carotid artery; IVT, intravenous thrombolysis; M1, M1 segment of middle cerebral artery; M2, M2 segment of middle cerebral artery; mRS, modified Rankin Scale; mTICI, modified Treatment in Cerebral Infarction score; PVT, prolonged venous transit; rCBF, relative cerebral blood flow; TIA, transient ischemic attack; Tmax, time to maximum.

### NIHSS scores stratified by the PVT status

Thirty‐six (30.3%) patients were PVT+. These patients had significantly more severe initial presentations reflected by higher admission NIHSS scores (17 (14–23.5) vs. 13 (9.5–19), *p* = 0.011) (Table 2). The discharge NIHSS scores were also significantly higher in PVT+ patients (7.5 (4–12) vs. 3 (1–7), *p* < 0.001). The NIHSS percent change was significantly lower in PVT+ patients (62 (21–79) vs. 74 (42–92), *p* = 0.020). Furthermore, the PVT+ patients were less likely to achieve a higher than 70% improvement in NIHSS scores (11 (30.6%) vs. 46 (55.4%), *p* = 0.013).

**Table 2:** NIHSS scores at admission and discharge and changes according to venous outflow dichotomized by prolonged venous transit.

	Total	Favorable venous outflow (PVT−)	Unfavorable venous outflow (PVT+)	*p* value
	*N* = 119	*N* = 83	*N* = 36	
Admission NIHSS	15 (11–20)	13 (9.5–19)	17 (14–23.5)	0.011
Discharge NIHSS	4 (2–10)	3 (1–7)	7.5 (4–12)	<0.001
∆NIHSS	9 (4–13)	9 (4–13)	9 (4–13)	0.772
NIHSS percent change	67 (33–88)	74 (42–92)	62 (21–79)	0.020
NIHSS percent change ≥70, *n* (%)	57 (47.9)	46 (55.4)	11 (30.6)	0.013

Values are represented as median (interquartile range) or number (percentage).

NIHSS, National Institution of Health Stroke Scale; PVT, prolonged venous transit.

### Determinants of NIHSS percent change

After adjusting for age, sex, hypertension, diabetes, atrial fibrillation, administration of IVT, ASPECTS score, mTICI 2c or 3, Tmax >6 s volume, and hemorrhagic transformation, PVT+ was independently associated with lower NIHSS percent change (*B* = −0.163, 95% CI −0.326 to −0.001, *p* = 0.049) and was less likely to achieve higher than 70% NIHSS improvement (OR = 0.331, 95% CI 0.127–0.863, *p* = 0.024) (Table 3). The additional significant predictor of less than 70% NIHSS improvement was hemorrhagic transformation (OR = 0.271, 95% CI 0.111–0.665, *p* = 0.004).

**Table 3:** Multivariable regression analysis for predictors of NIHSS percent change at discharge.

	NIHSS percent change^a^			NIHSS percent change ≥70^b^	
	Unstandardized coefficients B (95% CI)	Standardized coefficients *ß*	*p* value	Adjusted OR (95%CI)	*p* value
Age	−0.003 (−0.009, 0.002)	−0.130	0.210	0.980 (0.950, 1.011)	0.204
Female gender	0.008 (−0.150, 0.165)	0.010	0.923	1.194 (0.486, 2.934)	0.699
Hypertension	−0.033 (−0.225, 0.159)	−0.034	0.734	0.947 (0.316, 2.842)	0.923
Diabetes mellitus	−0.056 (−0.229, 0.118)	−0.063	0.527	1.201 (0.443, 3.252)	0.719
Atrial fibrillation	−0.035 (−0.193, 0.122)	−0.045	0.657	1.158 (0.467, 2.874)	0.752
ASPECTS	0.000 (−0.039, 0.038)	−0.002	0.983	1.096 (0.873, 1.376)	0.430
IVT	0.041 (−0.121, 0.202)	0.048	0.620	1.117 (0.434, 2.875)	0.819
mTICI 2c/3	0.119 (−0.047, 0.285)	0.135	0.158	2.119 (0.803, 5.591)	0.129
Tmax >6 s volume	0.000 (−0.002, 0.001)	−0.074	0.477	0.999 (0.992, 1.006)	0.788
Hemorrhagic transformation	−0.143 (−0.299, 0.012)	−0.181	0.070	0.271 (0.111, 0.665)	0.004
PVT+	−0.163 (−0.326, −0.001)	−0.190	0.049	0.331 (0.127, 0.863)	0.024

ASPECTS, Alberta Stroke Program Early CT Score; CI, confidence interval; IVT, intravenous thrombolysis; NIHSS, National Institution of Health Stroke Scale; OR, odds ratio; PVT, prolonged venous transit.

^a^ Multiple linear regression analysis was used.

^b^ Multivariable logistic regression analysis was used.

## Discussion

In this study of successfully reperfused AIS‐LVO patients, PVT+ was independently associated with a lower NIHSS percent change and a reduced likelihood of achieving more than 70% improvement in NIHSS scores. These findings provide supportive evidence that VO impairment, as represented by PVT+, is a key determinant of short‐term neurological changes in AIS‐LVO patients. To our knowledge, this is the first study to report associations between VO and NIHSS percent change.

The NIHSS is recognized as a reliable indicator of hospital length of stay, functional outcome at discharge, discharge disposition, and in‐hospital death.^16^, ^17^, ^18^, ^19^ As it is routinely applied upon admission to measure neurological deficits, it is also considered a valuable tool for tracking neurological functional changes over time. However, the use of absolute NIHSS score change has a major limitation: a specific absolute change might indicate substantial improvement in patients with lower baseline NIHSS scores, while the same change might not significantly impact functional outcomes in patients with more severe strokes. The NIHSS percent change offers a more accurate measurement by expressing change relative to the admission NIHSS score.^20^

Our study found that favorable VO was associated with lower admission NIHSS scores, consistent with previous research.^22^ Additionally, favorable VO was associated with a higher NIHSS percent improvement. These findings support the utility of PVT as a valuable adjunct imaging biomarker derived from CTP for assessing VO profiles in AIS‐LVO. PVT has already demonstrated its utility in predicting 90‐day functional outcomes^14^, ^15^ and this study further validates its association with short‐term neurological changes.

Detection of PVT+ is notably more discernible on post‐processed Tmax maps, aiding interpretation even for non‐experts. The cortical vein opacification scores (COVES) scale is a 6‐point system that requires evaluation of three specific veins (vein of Labbe, sphenoparietal sinus, and superficial middle cerebral vein).^7^ COVES has been served as a CTA imaging surrogate of VO assessment. A favorable VO has been associated with successful first‐pass reperfusion in AIS‐LVO,^5^ as well as with positive 90‐day functional outcomes following MT for anterior circulation LVO.^23^ Conversely, unfavorable VO correlates with a higher incidence of parenchymal hemorrhage,^24^ early brain edema,^2^ and poorer 90‐day outcomes.^24^ PVT is a binary parameter based on the assessment of two locations: the superior sagittal sinus (SSS) and the torcula. Compared to COVES, this approach simplifies the evaluation process, making it more practical for clinical settings. The superior sagittal sinus is indicative of superficial cerebral venous drainage while the torcula reflects deep venous drainage. Moreover, PVT+ is defined by the presence of prolonged perfusion time (Tmax ≥10 s), providing temporal information indicative of a severe delay relative to normal transit times. This dual aspect enhances its diagnostic value by capturing both static and dynamic aspects of venous circulation dysfunction in AIS‐LVO. Importantly, the PVT status is determined solely by the presence of Tmax ≥10 s and is unrelated to the volume of Tmax >10 s (Fig. 1).

Our study adds to the growing body of literature highlighting the significance of VO in the microperfusion of ischemic brain tissue. We demonstrated that VO is associated with less neurological deficit improvement during hospitalization. Prior research by Heitkamp et al. suggested that unfavorable VO is a strong predictor of unfavorable functional outcomes despite successful recanalization.^1^, ^9^ Adusumilli et al. further illustrated that a favorable comprehensive venous profile is strongly associated with functional independence and excellent postthrombectomy reperfusion.^11^ Additionally, Faizy et al. found that favorable VO correlates strongly with favorable tissue‐level collaterals indicated by HIR <0.4, ultimately leading to better functional outcomes.^25^, ^26^ Our findings are in agreement with these observations, further corroborating the growing evidence demonstrating the influence of VO for prognostication.

Our study found that hemorrhagic transformation (HT) was associated with a reduced likelihood of achieving more than 70% improvement in the NIHSS score. HT is a common complication of acute ischemic stroke and is linked to poor clinical outcomes.^27^ Previous research has shown that HT correlates with worse functional outcomes at discharge.^28^ Specifically, parenchymal hematoma type 2 (PH2) has been associated with diminished neurological improvement following thrombolysis (Gill, 2016).^29^ Unfavorable VO has been reported as a risk factor for hemorrhagic transformation, particularly parenchymal hematoma, after endovascular treatment.^6^, ^24^ In our cohort, while there was a higher incidence of PH2 (4.8% in PVT− vs. 13.9% in PVT+), the difference did not reach statistical significance, likely due to the low overall incidence of PH2 in our sample.

We acknowledge several limitations. Firstly, the retrospective design introduces inherent limitations such as selection bias and confounding factors. Additionally, the availability of CTP may be limited in smaller centers and rural locations, potentially impacting the generalizability of our findings. PVT is also subject to various factors, including the influence of blood flow, drainage patterns from non‐acutely affected regions, and aspects of the imaging protocol such as the rate of contrast injection and bolus timing. Moreover, the PVT in our study was designed to assess only two representative locations for simplicity, potentially limiting its comprehensive representation and susceptibility to local pathologies like thrombosis. Given these limitations, future research should aim for multicenter, prospective studies to further investigate the strength of the association between VO profiles and stroke outcomes, as well as to assess the generalizability of our findings across diverse patient populations and settings.

## Author Contributions

J.M, H.S, D.L, L.L, A.B, M.S, N.H, F.D, A.D, A.G, V.V, V.U, E.M, H.L, R.X, R.L, D.W, G.S, B.P, K.N, G.A, M.W, J.H, T.F, A.H, R.L, V.Y. contributed to the conception and design of the work. J.M, H.S, D.L, L.L, A.B, M.S, N.H, F.D, A.D, A.G, V.V, V.U, E.M, H.L, R.X, R.L, D.W, G.S, B.P, K.N, G.A, M.W, J.H, T.F, A.H, R.L, V.Y. were involved in the acquisition of data, and data analysis and interpretation. J.M, H.S, D.L, L.L, A.B, M.S, N.H, F.D, A.D, A.G, V.V, V.U, E.M, H.L, R.X, R.L, D.W, G.S, B.P, K.N, G.A, M.W, J.H, T.F, A.H, R.L, V.Y. drafted the work and revised it critically for important intellectual content. V.Y: Guarantor

## Conflict of Interest

The authors have no conflict of interest to declare.

## Funding Information

No funding information provided.

## Data Availability

The data supporting this study's findings are available from the corresponding author upon reasonable request.
